# Three-Dimensional Pharmacophore Design and Biochemical Screening Identifies Substituted 1,2,4-Triazoles as Inhibitors of the Annexin A2–S100A10 Protein Interaction

**DOI:** 10.1002/cmdc.201200107

**Published:** 2012-05-29

**Authors:** Tummala R K Reddy, Chan Li, Peter M Fischer, Lodewijk V Dekker

**Affiliations:** a School of Pharmacy, Centre for Biomolecular Sciences, University of NottinghamUniversity Park, Nottingham, Nottinghamshire NG7 2RD (UK); b Division of Pharmaceutical Chemistry, School of Pharmacy, Hertfordshire UniversityHatfield, Hertfordshire AL10 9AB (UK)

**Keywords:** drug design, pharmacophores, protein–protein interactions, structure–activity relationships, virtual screening

## Abstract

**Abstract:**

Protein interactions are increasingly appreciated as targets in small-molecule drug discovery. The interaction between the adapter protein S100A10 and its binding partner annexin A2 is a potentially important drug target. To obtain small-molecule starting points for inhibitors of this interaction, a three-dimensional pharmacophore model was constructed from the X-ray crystal structure of the complex between S100A10 and annexin A2. The pharmacophore model represents the favourable hydrophobic and hydrogen bond interactions between the two partners, as well as spatial and receptor site constraints (excluded volume spheres). Using this pharmacophore model, UNITY flex searches were carried out on a 3D library of 0.7 million commercially available compounds. This resulted in 568 hit compounds. Subsequently, GOLD docking studies were performed on these hits, and a set of 190 compounds were purchased and tested biochemically for inhibition of the protein interaction. Three compounds of similar chemical structure were identified as genuine inhibitors of the binding of annexin A2 to S100A10. The binding modes predicted by GOLD were in good agreement with their UNITY-generated conformations. We synthesised a series of analogues revealing areas critical for binding. Thus computational predictions and biochemical screening can be used successfully to derive novel chemical classes of protein–protein interaction blockers.

## Introduction

Protein–protein interactions are widely regarded as pivotal to cell regulation and are increasingly of interest as targets in small-molecule drug discovery.^1^ Disruption of protein–protein interactions can be challenging; however, some progress has been made in this area. For example, the interaction between p53 and MDM2, which involves a comparatively small binding interface, can be blocked by several chemical classes of small molecules, some of which have progressed to the clinical investigation stage.^2^ Similarly, small-molecule BH3 domain mimetics block the interaction of anti-apoptotic Bcl2 family members with BH3-domain-containing pro-apoptotic proteins.^3^ These compounds show tumour growth inhibition in tumour models and have progressed to early clinical-stage research.^3^ Finally, various investigational small-molecule blockers have been reported for the protein interaction between VLA4–VCAM,^4^ B7.1–CD28,^5^ oestrogen-related receptor-α and its co-activator,^6^ and protein kinase C-iota and its effector PAR6.^7^

The interaction between S100 proteins and their targets has been implicated in numerous biological and pathological processes. The principle function of S100 proteins is regulation of the localisation and activity of other proteins by direct protein–protein interactions. Some of these interactions have potential therapeutic relevance. Recent genetic deletion studies have implicated both S100A10 and its binding partner annexin A2 in the process of neo-angiogenesis.^8^ This parallel phenotype suggests these two partners may form a physical complex to regulate this process. Small-molecule blockers of the interaction would greatly aid the further investigation of this idea. At the molecular level, the interaction is very well characterised both by mutagenesis and crystallography, involving a small and largely hydrophobic binding area.^9^ Two S100A10 molecules form a dimeric structure that yields two binding pockets, each of which accommodates the 14-residue N-terminal region of annexin A2. Using a structure-based virtual high-throughput docking approach, we previously identified several clusters of small molecules that dock into the annexin A2 binding pocket on S100A10.^10^ Biochemical screening of these showed that substituted 4-aroyl-3-hydroxy-5-phenyl-1*H*-pyrrol-2(5*H*)-one analogues are capable of inhibiting the binding between S100A10 and annexin A2.^10^ In the present study we sought to provide further evidence that this protein interaction can be targeted with small-molecule blockers. To do so, we used a ligand-guided method as an alternative to the above random docking approach. The binding pose of the cognate annexin A2 N terminus into the S100A10 dimer is very well characterised, and we used the topological arrangement of key chemical features in the annexin A2 N terminus known to be important for interacting with the S100A10 protein, to design a 3D pharmacophore. We screened a library of compounds against this 3D pharmacophore and identified molecules that match these features and their topology. These were tested for inhibition of the interaction between S100A10 and the annexin A2 N terminus. Thus we established that substituted 1,2,4-triazole analogues are able to compete with the binding of the annexin A2 N terminus to S100A10.

## Results

### 3D pharmacophore generation

**Figure 1:**
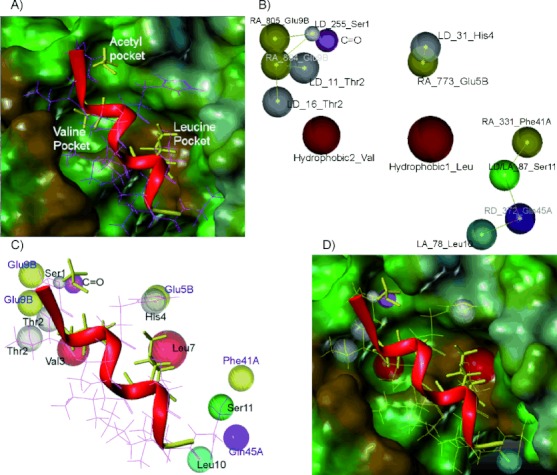
A) Binding pose of the annexin A2 N-terminal peptide (represented as lines with the secondary helical structure superimposed) within the binding site of S100A10. The isopropyl side chain of Val3 (yellow sticks) is directed into the valine pocket. The side chain of Leu7 (yellow sticks) partly occupies the leucine pocket. The acetyl group at Ser1 (yellow sticks) occupies the acetyl pocket. B) Schematic representation of the pharmacophoric query. Two hydrophobic features (Hydrophobic1_Leu, Hydrophobic2_Val) are shown as red spheres, whilst the carbonyl group (C–O, Markush atom) is represented by a magenta sphere. Hydrogen bonds are defined as vectors (shown as yellow lines) from the donor atom of the peptide to the corresponding acceptor atom in the receptor and vice-versa. Asterisks indicate the centres of the spheres. Hydrogen bond donor features of the peptide (LD_16_Thr2, LD_11_Thr2, LD_255_Ser1, LD_31_His4) are shown as grey spheres. Hydrogen bond acceptor features of the receptor (S100A10) are shown in yellow (RA_805_Glu9B, RA_804_Glu9B, RA_773_Glu5B, RA_331_Phe41A). For Ser11 of the peptide, the side chain OH group acts as hydrogen bond donor as well as hydrogen bond acceptor feature (LD/LA_87_Ser11), represented as a green sphere. The hydrogen bond donor atom feature (RD_372_Gln45A) of the receptor is shown as an indigo sphere. The hydrogen bond acceptor feature (LA_78_Leu10) of the peptide is shown as a cyan sphere. For clarity, the receptor site constraints are not shown in the query (see also panel D). C) Overlay of the pharmacophore query generated under B and the annexin N-terminal peptide in its docked pose as shown under A. Amino acids associated with the pharmacophoric features are indicated in blue (receptor features) or black (ligand features). D) Representation of the pharmacophore query in the annexin N terminus binding groove of S100A10. The images in panels A and D were generated using SYBYL-X Molcad with the molecular surface of S100A10 coloured according to lipophilic potential. Images in panels B and C were generated with the SYBYL-X UNITY module.

Taking into account the crystal structure model of the complex between S100A10 and the annexin A2 N terminus and the effect of modification of the annexin A2 N terminus on its interaction with S100A10,^9^ a pharmacophoric query was constructed. Within the annexin A2 N terminus, the valine residue at position 3 and the leucine residues at positions 7 and 10 are crucial for binding with S100A10 as is the N-acetyl group of the N-terminal serine residue. Contact surface analysis indicates that the acetyl group, as well as the Val3 and Leu7 residues point into the pocket (Figure 1 A, represented as yellow sticks). The acetyl group occupies a relatively small hydrophilic pocket (acetyl pocket, Figure 1 A) composed of Pro1, Ser2, His6, and Glu9 of molecule B of the S100A10 dimer. The isopropyl side chain of Val3 is accommodated in a hydrophobic pocket, which is also solvent accessible and is composed of Cys82 of S100A10 molecule A, and Met8, Met12, and Glu9 of S100A10 molecule B (valine pocket, Figure 1 A). The side chain of Leu7 also partly occupies another hydrophobic cavity composed of Phe38, Pro39, Gly40, Phe41, and Leu78 of S100A10 molecule A, and Glu5 and Met8 of S100A10 molecule B (leucine pocket, Figure 1 A).

The above hydrophobic contacts of Val3 and Leu7 are represented by hydrophobic features in the pharmacophore query (Figure 1 B–D) and include six- and five-membered rings, *tert*-butyl, cyclopropyl, isobutyl, and other aliphatic chains. Based on the size of the cavity available within the binding pocket, a spatial constraint with a tolerance of 1.5 Å was added to the Leu7 hydrophobic feature, and a spatial constraint with a tolerance of 1.25 Å was added to the Val3 hydrophobic feature. This resulted in spherical hydrophobic features (red, Figure 1 B–D) reflecting the tolerance in the 3D position. A carbonyl group, representing the N-acetyl group of annexin A2, was defined as a third pharmacophoric feature. Because the relevant pocket is relatively small, a spatial point constraint with a tolerance of 0.75 Å was added to the carbon atom (magenta sphere, Figure 1 B–D). This feature was further enhanced by employing Markush carbonyl atom definition, which allows compounds with specific functional groups at the acetyl pocket. These include -COCH_3_, -CONH_2_ -COCH_2_CH_3_, -COOCH_3_, -COOCH_2_CH_3_, -NHCOCH_3_, -NHCOCH_2_CH_3_, -NHCOOCH_3_, -NHCOOCH_2_CH_3_, -NHCONH_2_, -CH_2_COOH, and -NHCOOH.

Hydrogen bond interactions are represented in the query in the form of hydrogen bond donor and acceptor features (Figure 1 B–D). The side chain hydroxy group (LD_16_Thr2, grey sphere) and the backbone NH group (LD_11_Thr2, grey sphere) of Thr2 undergo hydrogen bond donor interactions with the carboxyl group (RA_804_Glu9B, yellow sphere) of the Glu9 residue in S100A10 molecule B. The backbone NH group of Ser1 (LD_255_Ser1, grey sphere) forms hydrogen bond donor interactions with the side-chain carboxyl (RA_805_Glu9B and RA_804_Glu9B yellow spheres) of Glu9 in S100A10 molecule B. The ring NH group of His4 (LD_31_His4, grey sphere) forms a hydrogen bond donor interaction with the carboxyl group (RA_773_Glu5B, yellow sphere) of Glu5 in S100A10 molecule B. Furthermore, the side-chain hydroxy group (LD_87_Ser11, green sphere) of Ser11 forms a hydrogen bond donor interaction with the backbone carboxyl oxygen (RA_331_Phe41A, yellow sphere) of Phe41 in S100A10 molecule A and hydrogen bond acceptor interaction (LA_87_Ser11, green sphere) with the side chain carboxamide NH_2_ (RD_372_Gln45A, indigo sphere) of Gln45 in S100A10 molecule A. The backbone carbonyl oxygen (LA_78_Leu10, cyan sphere) of Leu10 undergoes a hydrogen bond acceptor interaction with the side-chain carboxamide NH_2_ (RD_372_Gln45A, indigo sphere) of Gln45 in S100A10 molecule A. A spatial constraint with a tolerance of 1.0 Å was added to all hydrogen bond donor and acceptor features except for the hydrogen bond donor feature of Ser1, for which a tolerance of 0.25 Å was added in order to avoid the overlap of its spatial constraint with the adjacent carbonyl atom spatial constraint. A final significant feature included in the query was the receptor site constraint. This was added in the form of multiple excluded volume spheres with a van der Waals scaling factor of 0.5 Å (not shown for clarity). For a compound to be considered as a hit, it should match the query features without colliding with multiple excluded volume spheres that represent the protein surface.

### Computational screening for pharmacophore matches

A 3D database containing 704 511 structurally diverse compounds was generated from the Zinc database^11^ (Asinex and ChemBridge vendors) using the following five molecular filters: 1) *M*_r_ range: 150–600 Da, 2) hydrogen bond donors: <7, 3) hydrogen bond acceptors: <14, 4) Xlog *P*: <5, and 5) rotatable bonds: <12. For each molecule, a single conformation was stored in the 3D database. A UNITY flexible 3D search was then performed on the 3D pharmacophore model using the direct tweak algorithm, which adjusts the rotatable bonds of the molecules to match the 3D pharmacophoric model as closely as possible. The search was designed to match the most significant hydrophobic features derived from the leucine and valine pockets as well as the Markush carbonyl definition with a receptor site constraint in place. In addition, the search was refined to allow partial matching of hydrogen bonding donor or acceptor features, such that for a molecule to be considered a hit, a minimum of one and a maximum of five hydrogen bonding features were required to fit. The entire UNITY 3D database search of the 704 511 compounds was performed directly on an Intel I7-based LINUX system. The time taken for the search was ∼120 h. This search returned 568 hits.

These hits were ranked based on their best-fit values generated from the 3D search. The fit values reflect the agreement of the hit compounds with the pharmacophore model, with higher fit values indicating better mapping of the hit compound onto the pharmacophore model. The fit values ranged from 62 to 8. The hit compounds were also docked using the Genetic Optimisation for Ligand Docking (GOLD) program^12^ in standard parameter mode into the defined annexin A2 binding pocket of the S100A10 receptor. Docked compounds were ranked based on the GOLD score, which represents the sum of receptor–ligand hydrogen bonding energy, van der Waals energy, torsional energy, and hydrophobic interaction energies and ranged from 66 to 36. Higher GOLD scores indicate better binding interaction of the compounds with the S100A10 receptor. The docking procedure for the 568 hit compounds was carried out using the GOLD (V3.0.1) docking program; calculations required ∼1 h running on 32 dual AMD Opteron 248 servers. A total of 190 hits that were selected based on best-fit values, higher GOLD fitness scores, and a good binding mode in accordance with our pharmacophore query were purchased.

### Identification and analysis of inhibitory compounds

**Figure 2:**
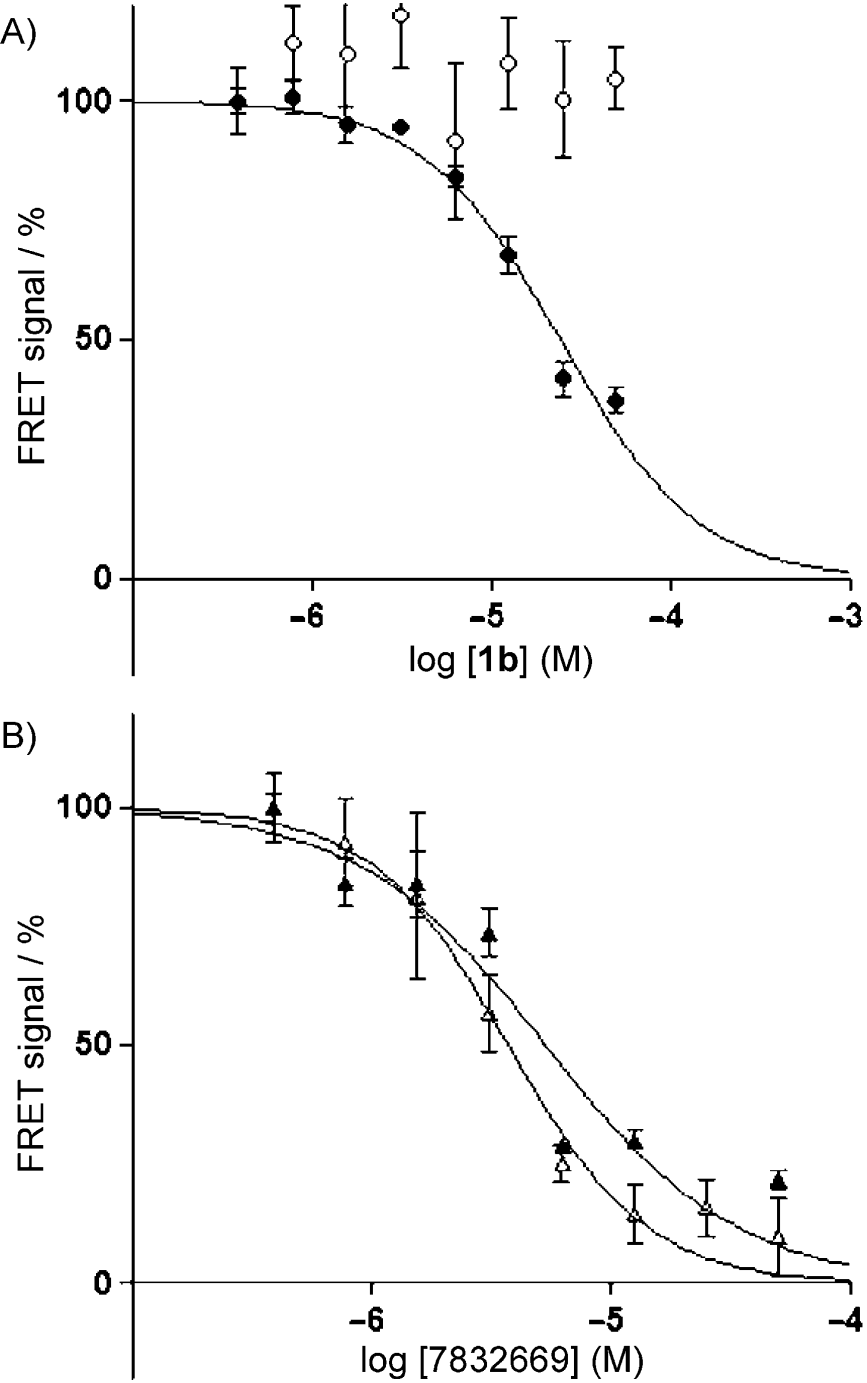
Inhibitory activity of A) compound 1 b and B) compound 7832669 (ChemBridge reference number; also referred to as ZINC02858054). Both compounds were tested for inhibition of the interaction between S100A10 and the annexin A2 N terminus (• and ▴) or for inhibition of a non-target protein interaction (○ and ▵) as explained in the Results and Experimental Section. Data points represent the average ±SEM of four observations.

**Figure 3:**
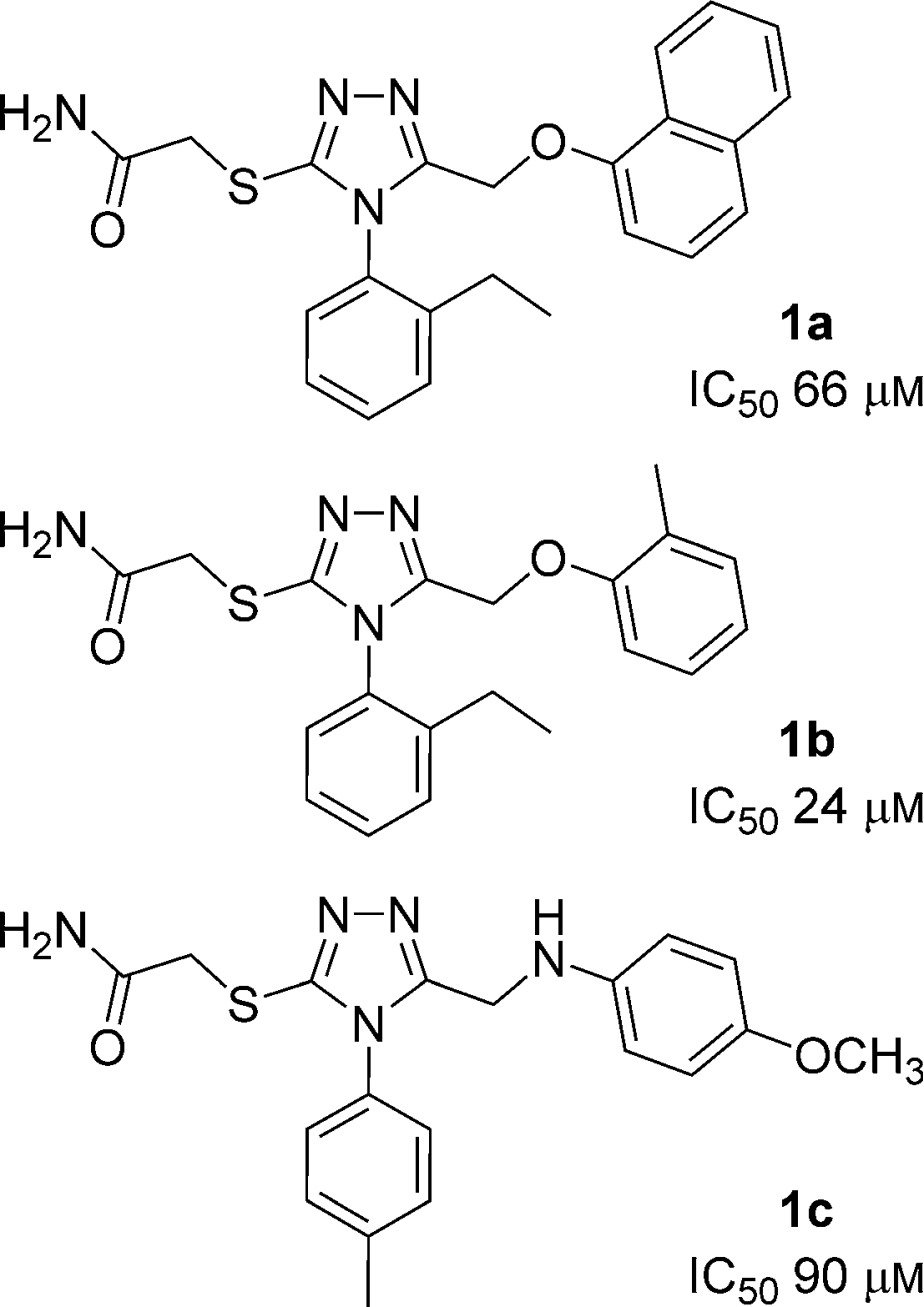
Structures and IC_50_ values of hit compounds.

**Figure 4:**
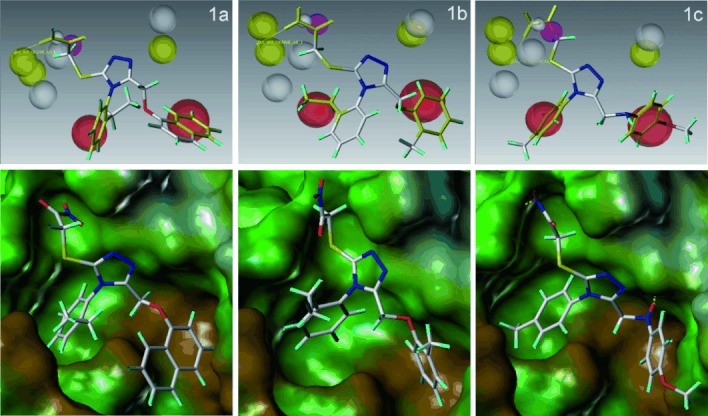
Top panels: Mapping of the 3D pharmacophore query onto the inhibitors (1 a–c); the fit values were 30, 20, and 25, respectively. The compounds showed good agreement between their UNITY-generated conformations and the conformations of the binding pose predicted by GOLD (bottom panels). GOLD scores of compounds 1 a–c were 54, 51, and 54, respectively. Mapping was carried out using the SYBYL-X UNITY module, and the binding poses were generated with the GOLD docking program. The molecular surface of the S100A10 is coloured according to lipophilic potential.

Hits were tested for the inhibition of the interaction between S100A10 and the annexin A2 N terminus in a competitive fluorescent binding assay.^13^ Each compound was tested in quadruplicate at a single concentration of 10 μm. Compounds showing inhibitory activity (defined as signal deviating by >3 standard deviations from the no-compound control) were selected for repeat testing at the same concentration. Seven compounds showed consistent levels of inhibition, and these were taken forward for IC_50_ determination. Furthermore, these compounds were analysed in a counterscreen, which measures a nonrelated protein interaction using the same assay format. Three compounds showed activity in the primary assay, but not in the counterscreen assay (Figure 2 A), whilst four compounds showed overlap in activity between the two assays (Figure 2 B for an example: a compound from the ChemBridge catalogue with vendor number 7832669 and Zinc database number ZINC02858054). The latter compounds could represent promiscuous protein interaction blockers, or compounds that in some way interfere with the fluorescence signal. The structures of three confirmed hit compounds are shown in Figure 3. Interestingly, all hit compounds featured the same acetamide side chain, suggesting a potentially significant contribution of this group to binding interactions. Analysis of the mapping of the pharmacophore onto these inhibitors indicates that the hydrophobic feature associated with the leucine pocket mapped onto one of the phenyl rings of the naphthyl ring system (**1 a**), onto the phenyl moiety of the *ortho*-toluyl group (**1 b**), or the phenyl moiety of the *para*-anisole group (**1 c**) (Figure 4, top panels). The hydrophobic feature associated with the valine pocket mapped onto the N4-phenyl rings in compounds **1 a** and **1 c**, whereas in the case of compound **1 b** it overlapped with the *ortho*-ethyl group of the N4-phenyl ring. In the case of compounds **1 a**, **1 b**, and **1 c** the amide group matched the Markush atom definition with the NH_2_ group locating to the hydrogen bond donor feature, within hydrogen bonding distance to the carboxyl group of Glu9 in S100A10 molecule B. Importantly, the UNITY-generated conformations of compounds **1 a**, **1 b**, and **1 c** mapped onto the 3D pharmacophoric query without clashing with the receptor site (multiple excluded volume spheres).

The binding modes predicted by GOLD for compounds **1 a**, **1 b**, and **1 c** are in good agreement with their UNITY-generated conformations (Figure 4, bottom panels). The predicted binding modes indicate that the substituted aromatic rings closely mimic Val3 and Leu7 in annexin A2, interacting with the hydrophobic pockets of S100A10. In case of compounds **1 a** and **1 b** the NH_2_ group of the amide side chain at the acetyl pocket is in close proximity to Glu9 in S100A10 molecule B, suggesting the possibility of hydrogen bond interactions taking place. In compound **1 c** both the NH_2_ group of the amide and the secondary amine group undergo hydrogen bond interactions with the carboxyl groups of S100A10 B-chain Glu9 and S100A10 B-chain Glu5, respectively. This appears to mimic the hydrogen bond interaction of Glu9 and Glu5 residues of the receptor with the annexin A2 N-terminal peptide in the crystal structure. Considering the inhibitory potential of these triazole compounds **1 a** (66 μm), **1 b** (24 μm), and **1 c** (90 μm) we synthesised a series of analogues and investigated their structure–activity relationships.

### Synthesis of substituted 1,2,4-triazoles

**Scheme 1:**
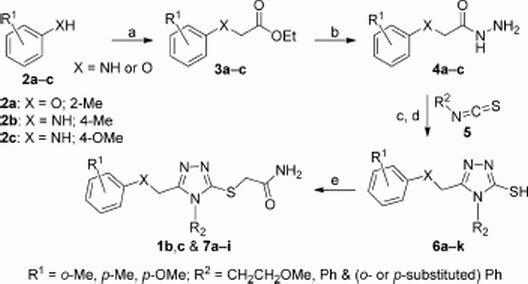
Synthesis of 2-(4-substituted-5-substituted phenyl oxy/amino methyl-4*H*-[1,2,4]triazol-3-ylsulfanyl)acetamides. *Reagents and conditions*: a) X=O: DMF, under N_2_, NaH, 60 min, RT followed by bromoethyl acetate, 30 min, RT, then H_2_O; X=NH: bromoethyl acetate, Et_3_N, EtOH, reflux, 30–75 min; b) H_2_NNH_2_⋅H_2_O, EtOH, reflux, 5 h to overnight; c) EtOH/reflux, overnight; d) 1.5 m KOH (base-catalysed cyclisation), 5 n HCl; e) 2-bromoactamide, K_2_CO_3_, DMF, 45 °C, overnight, then H_2_O.

The required disubstituted 1,2,4-triazoles **1 b**, **1 c**, and **7 a**–**i** were prepared as depicted in Scheme 1. The substituted acetic acid ethyl ester **3 a** (X=O) was prepared by treating *ortho*-cresol **2 a** with bromoethyl acetate in the presence of sodium hydride in *N*,*N*-dimethylformamide. The substituted acetic acid ethyl esters **3 b**–**c** (X=NH) were prepared by treating substituted anilines **2 b**–**c** with bromoethyl acetate in the presence of triethylamine in ethanol. Subsequent reaction of ethyl esters **3 a**–**c** (X=O or NH) with hydrazine monohydrate gave the corresponding acyl hydrazides **4 a**–**c** in good to excellent yields.^14^ Condensation of the acyl hydrazides **4 a**–**c** with substituted aromatic or aliphatic isothiocyanates **5**, followed by base-catalysed cyclisation, resulted in 4-substituted 3-mercapto-1,2,4-triazole analogues **6 a**–**k**. Treatment of these intermediates with 2-bromoacetamide in the presence of potassium carbonate resulted in the formation of disubstituted 1,2,4-triazole analogues **1 b**–**c** and **7 a**–**i**.^15^

### Structure–activity relationships

The 1,2,4-triazole compounds synthesised were screened using an in vitro FRET-based competitive binding assay to evaluate their ability to inhibit the interaction between S100A10 and an annexin A2 N-terminal peptide.^13^ An IC_50_ value was obtained for each compound from nonlinear regression analysis (GraphPad Prism, variable slope) with top and bottom constraints of 100 and 0 %, respectively.

To investigate the contribution of the phenyl ring at the N4 position of the 1,2,4-triazole ring system to the inhibition of binding, a set of analogues with X=O (**7 a**–**g**) was prepared (Table 1). An unsubstituted phenyl ring at the N4-position (**7 a**, IC_50_=230 μm) showed very low potency, suggesting that the aromatic ring alone is insufficient for inhibitory activity and that the substitution pattern is important. Replacement of the N4 ring system with a flexible aliphatic methoxyethyl group (yielding **7 c**) abolished activity altogether, reinforcing the notion that a substituted aromatic ring system at this position is important for inhibition of binding. With reference to compound **7 a** (230 μm), the effect of various substitutions on the phenyl ring was assessed. An *ortho*-ethyl substitution (**1 b**, 24 μm) showed increased potency, possibly due to the introduction of a conformational restriction imposed on the phenyl group. However, introduction of a bulky chloro group at the *ortho* position (**7 b**, 176 μm) was less effective in terms of inhibitory activity. Introduction of a *para*-methyl group (**7 d**, 88 μm) showed a moderate increase in inhibitory activity, but a chloro group at the *para* position (**7 e**, 710 μm) resulted in a profound loss of activity. Introduction of a more polar methoxy substituent at the *para* position (**7 f**, 122 μm) did not affect the inhibition of binding, but a compound with an isopropyl side chain at the *para* position (**7 g**, 27 μm) was comparatively potent. This may be due to the fact that the phenyl ring projects towards the valine pocket, with the isopropyl group mimicking the valine side chain of the annexin peptide.

**Table 1:** Structure–activity relationships of 2-(4-substituted-5-substituted phenyl oxy/amino methyl-4*H*-[1,2,4]triazol-3-ylsulfanyl)acetamides.

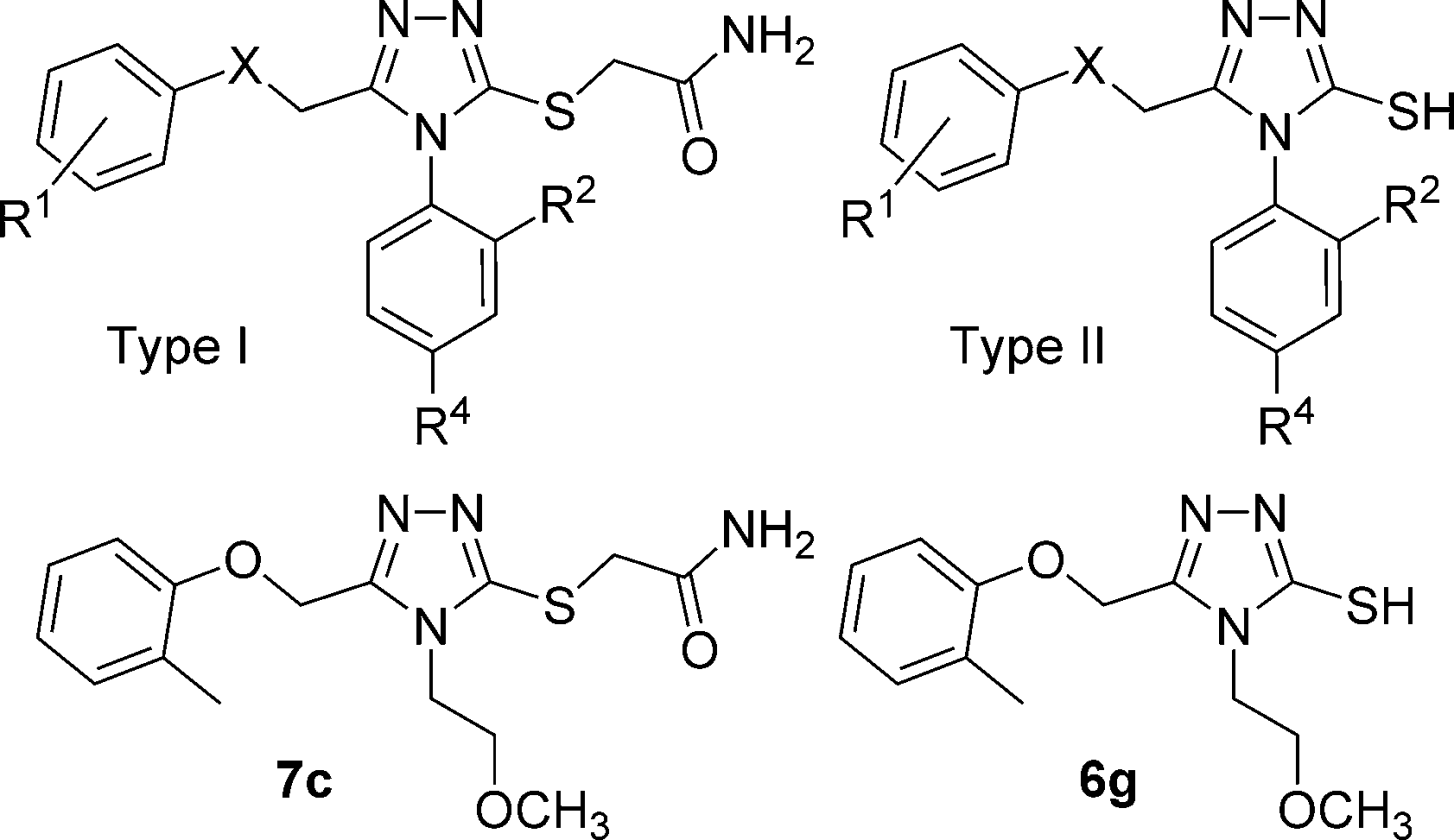						
Compd	Type	X	R^2^	R^4^	R^1^	IC_50_ [μm]^[a]^
**7 a**	I	O	H	H	2-Me	230
**1 b**	I	O	Et	H	2-Me	24
**7 b**	I	O	Cl	H	2-Me	176
**7 c**	–	–	–	–	–	NA
**7 d**	I	O	H	Me	2-Me	88
**7 e**	I	O	H	Cl	2-Me	710
**7 f**	I	O	H	OMe	2-Me	122
**7 g**	I	O	H	*i*Pr	2-Me	27
**7 h**	I	NH	H	Cl	4-Me	NA
**1 c**	I	NH	H	Me	4-OMe	90
**7 i**	I	NH	Et	H	4-OMe	75
**6 a**	II	NH	Et	H	4-OMe	NA
**6 b**	II	NH	H	Me	4-OMe	35
**6 c**	II	NH	H	Cl	4-Me	115
**6 d**	II	O	H	H	2-Me	481
**6 e**	II	O	Et	H	2-Me	275
**6 f**	II	O	Cl	H	2-Me	83
**6 g**	–	–	–	–	–	117
**6 h**	II	O	H	Cl	2-Me	23
**6 i**	II	O	H	Me	2-Me	partial
**6 j**	II	O	H	OMe	2-Me	partial
**6 k**	II	O	H	*i*Pr	2-Me	68

[a] NA: no inhibition was observed; partial: compound behaved as partial blocker of binding.

Because one of the original hit compounds (**1 c**) contains X=NH, a small set of analogues with X=NH were prepared, and substituents at the N4 and R^1^ positions of the 1,2,4-triazole ring system were varied (Table 1). Compound **1 c** (90 μm) with a *para*-methylphenyl substituent at the N4 position and a *para*-methoxy substituent at the R^1^ position showed weak inhibitory activity. Replacement of both *para*-methyl and methoxy substituents in **1 c** with chloro and methyl substituents (**7 h**) resulted in loss of activity. Replacement of the *para*-methyl group in **1 c** with a bulkier *ortho*-ethyl group (**7 i**, 75 μm) largely retained activity. These patterns are, to an extent, reminiscent of those observed in the X=O analogues, with a *para*-chloro substituent being highly disfavoured (**7 e**, 710 μm; **7 h**, inactive), and an *ortho*-ethyl group supporting inhibitory activity of these compounds (**1 b**, 24 μm; **7 i**, 75 μm). The NH group may act as a hydrogen bond donor to the carbonyl oxygen of glutamic acid of the S100A10 protein. Reference to the modelled binding pose of **1 c** (Figure 4), in which the NH group is hydrogen bonded to the carbonyl oxygen atom of glutamic acid of S100A10, supports this notion. In this regard, the loss in activity observed for compound **7 h** was unexpected.

As mentioned above, a carbonyl group (representing the peptide N-acetyl) was defined as the third pharmacophoric feature, and a range of various functional groups were allowed at this part of the query. Rather strikingly, all three hit compounds contained the same acetamide side chain mapping onto the pharmacophore, suggesting a potentially significant contribution of this group for binding interactions. This was investigated further by truncating the sulfanylacetamide side chain to the corresponding thiol. This resulted in a sixfold decrease in binding activity of compound **1 b**, 24 μm (**6 e**, 275 μm), a threefold decrease in binding activity of compound **7 g**, 27 μm (**6 k**, 68 μm) and a twofold further decrease in binding activity of the already very weak blocker **7 a**, 230 μm (**6 d**, 481 μm). For compounds **7 d**, 88 μm and **7 f**, 122 μm (yielding **6 i** and **j**, respectively), this modification resulted in conversion into partial blockers of the interaction, inhibiting <50 % of the binding. Thus the acetamide side chain appears to make an important contribution to the activity of these compounds, possibly by acting as a hydrogen bond donor to its receptor. Consistent with this trend, truncation of the sulfanyl acetamide side chain of compound **7 i** (75 μm) to the thiol **6 a** resulted in complete loss of activity. However, similar truncation compounds **7 c** and **7 h** (both inactive) resulted in moderately active compounds (**6 g**, 117 μm and **6 c**, 115 μm respectively), whilst this modification is associated with a small increase in potency for compound **1 c**, 90 μm (**6 b**, 35 μm) and a large gain in potency for **7 e**, 710 μm (**6 h**, 23 μm).

## Discussion

We have presented a ligand-based virtual screening approach to identify inhibitors of the protein interaction between annexin A2 and S100A10. Based on the binding pose of the annexin A2 N terminus in S100A10, a Markush carbonyl atom, hydrophobic and hydrogen bond interactions, along with spatial and receptor constraints, were established as key pharmacophoric features that were translated into a 3D pharmacophore. Screening of >700 000 compounds against this pharmacophore resulted in the identification of 1,2,4-triazole compounds **1 a**–**c** as novel genuine inhibitors of the S100A10–annexin A2 interaction. The GOLD-predicted binding modes of these compounds were analysed, revealing close similarity to the UNITY-predicted binding modes. A set of 22 compounds were synthesised, and SARs were explored. Four compounds (**1 b**, **7 g**, **6 b**, **6 h**) showed inhibitory activity <50 μm and another set of six compounds (**1 a**, **1 c**, **7 d**, **7 i**, **6 f**, **6 k**) showed inhibitory activity <100 μm. Overall, some interesting SAR patterns emerged, with hydrophobic features favoured at the N4-position of the 1,2,4-triazole ring system and an acetamide side chain making contributions to binding in the majority of compounds. Thus an approach involving generation of a pharmacophoric query based on the peptide binding pose of annexin A2 in the S100A10 dimer and subsequent biochemical screening of candidate pharmacophore matches, has yielded small-molecule blockers that could be developed further in terms of inhibitory potential and drug-like properties.

Previous work in our research group identified substituted pyrrol-2-ones as potential blockers of the interaction of annexin A2 with S100A10,^10^ using a random docking approach to computational pre-selection of the compounds that were tested biochemically. This approach interrogates compounds for their geometric complementarity with the target receptor binding pocket, whilst the 3D pharmacophore design method used in the current study selects compounds for similarities to specific chemico-topological features of the ligand that are associated with binding.^16^ For both methods, a static 3D co-crystal model9b was used as starting point for the design process. However, flexibility of the system will affect what the binding site will actually look like when the designed ligands meet the protein in the biochemical assay system.^16^ Uncertainties in the prediction could explain why different scaffolds were identified in the two studies, and suggests the usefulness of employing multiple strategies for hit finding, as previously suggested for other drug targets.^17^

Some of the pyrrol-2-ones^10^ appeared to be more potent than the most potent 1,2,4 triazoles described here. The reasons are unclear, and the number of compounds tested in either study precludes a generalised conclusion. Potency differences observed between the studies may be due to the way the parameters for curve fitting of the biochemical data were set, which differed slightly. However, reanalysis using the same parameter settings still showed the pyrrol-2-ones to be more potent than the 1,2,4 triazoles described herein.

There are other protein–protein interactions for which small-molecule blockers have been identified. Notable amongst these are two protein interactions implicated in cancer biology, namely the interaction between MDM2 and p53,^2^, ^18^ and the interaction between Bcl2 and Bak.^3^ Like annexin A2, p53 and Bak contain a small helical structure that docks into a well-defined groove-like feature on the surface of the respective binding partners. Thus whilst protein–protein interactions may be considered difficult targets for small-molecule inhibitors, this cannot be generalised, and certain interactions with shared features in terms of the binding site appear to be more amenable to small-molecule inhibition than others. For the interaction between S100A10 and annexin A2, the proposed 1,2,4-triazole compounds will be useful for further biological assessment of the function of this complex. Furthermore, they may be starting points for lead optimisation and further development.

## Experimental Section

### Computational techniques

**S100A10–annexin A2 N-terminal peptide complex**: The Tripos (SYBYL-X 1.0) Biopolymer module was used for the protein structure preparation process. Two chain termini (Lys91, Pro1) of each monomer of the S100A10 and Ser11 of each annexin A2 N-terminal peptide were charged. Hydrogen atoms were added, and Amber 7 FF99 charges were assigned to the complex. The side chain amides of Gln45 and Gln60 were re-oriented to maximise hydrogen bonding. The complex was then subjected to stepwise energy minimisation. The parameters used were Amber 7 FF99 force fields, Powell’s minimisation method, 0.5 kcal mol^−1^ gradient, no initial optimisation was performed, and a maximum of 5000 iterations were used. A centroid was defined by a set of atoms within 5 Å of the annexin A2 peptide, and the peptide was then extracted from the complex. Water molecules were deleted before docking.

**UNITY search**: The UNITY module of SYBYL-X (V1.0) was used for the design of a highly focused pharmacophoric query. The query was directly built in UNITY from the energy-minimised co-crystal structure of S100A10–annexin A2 N-terminal peptide as described in the results section, using relevant features (hydrogen bond acceptor atom, hydrogen bond donor atom, acceptor site, and donor site to specify the directionality of the hydrogen bond, hydrophobic features) and constraints (distance, spatial point, and receptor site constraints). UNITY flex searches were performed on 3D compound databases with a single conformational representation of each compound, using the direct tweak algorithm to match the conformation of the molecule with the defined pharmacophoric query. Hits were generated based on how well the compounds matched the query.

**GOLD (V3.0.1) docking studies**: Ten docking runs were performed on each molecule and allowed the early termination of the docking runs if the top three solutions were within the 1.5 Å RMSD of each other. During the run, 100 000 genetic algorithm (GA) operations were performed on a single population of 100 individuals. Operator weights for the crossover, mutation, migration (95, 95, and 10 respectively) hydrogen bonding (4.0 Å), and van der Waals (2.5 Å) parameters were set as default value throughout the docking. An active site radius value of 12 Å was found to be optimum. Flipping was not allowed for those ligands that have ring-NHR and ring-NHR^1^R^2^ groups in order to avoid the addition of large torsional energy penalties to the total fitness scores.

### Biology

*Fluorescence screening assay*: Routine assessment of compound activity was performed as described by Li et al.^13^ Briefly, a Cy5-labelled S100A10 tracer was developed, and binding of a Cy3-labelled annexin A2(1–14) peptide ligand was assessed using a fluorescence resonance energy transfer (FRET) readout. Assays were carried out in Nunc black non-treated 384-well plates at 20 °C in 50 μL 137 mm NaCl, 2.7 mm KCl, 4.3 mm Na_2_HPO_4_, 1.47 mm KH_2_PO_4_ pH 7.4 containing 2 mm 1,4-dithiothreitol. All incubations were performed in quadruplicate. Compounds, peptide, and buffer controls were added to the wells in a 10 μL volume in 5 % DMSO. Cy5-labelled S100A10 tracer (407 nm) and Cy3-labelled annexin A2(1–14) peptide ligand (1.33 μm) were pre-incubated for 5 min at 20 °C, and 40 μL of the preformed complex was then added to the wells and mixed for 10 s to yield a final DMSO concentration of 1 %. After 5 min incubation at 20 °C, readings were taken on a PerkinElmer Envision instrument by excitation at 488 nm and emission at 695 nm. As controls, each screening plate contained the individual labelled proteins, as well as the two protein partners with and without the unlabelled annexin A2(1–14) competitor (12.8 μm; all in quadruplicate).

FRET was calculated by measuring the fluorescence emission of co-incubated S100A10–Cy5 and annexin A2(1–14)–Cy3 and subtracting the same signal obtained in the presence of excess unlabelled annexin A2(1–14) peptide. A counterscreen assay,^13^ which measures the FRET signal from a Cy3-conjugated donkey anti-goat IgG (4 μg mL^−1^) onto a Cy5-labelled goat IgG (3 μg mL^−1^), was used in parallel to assess nonspecific interference with the fluorescence readout. Compound binding was calculated as a percentage of untreated control, and data were analysed by nonlinear regression (dose–response, variable slope) using GraphPad Prism Software with top and bottom constraints at 100 and 0 %, respectively (unless otherwise discussed in the text).

### Chemistry

**General**: All reagents were purchased directly from commercial sources and were used as supplied unless otherwise stated. Melting points were measured with a Gallenkamp melting point apparatus and are uncorrected. Accurate mass and nominal mass measurements were performed using a Waters 2795-Micromass LCT electrospray mass spectrometer. Infrared spectra were recorded on an AVATAR 360 FTIR system. Samples were prepared as KBr discs and scanned from 4000 to 500 cm^−1^ (for compounds **7 i** and **6 a**). All NMR spectra were recorded in [D_6_]DMSO, with trimethylsilane as an internal standard, using a Bruker ACS-120 instrument at 400 MHz (^1^H NMR) and 100.6 MHz (^13^C NMR). Chemical shifts (*δ*) are reported in ppm, and coupling constants (*J*) are given in Hz. Signals are represented by s (singlet), d (doublet), t (triplet), q (quartet), m (multiplet), bs (broad singlet), dd (double doublet), and td (triple doublet). Thin-layer chromatography was performed using aluminum-backed silica gel 60 plates (0.20 mm layer), the ascending technique was used with a variety of solvents. Visualisation was by UV light at either *λ* 254 or 365 nm.

***o*****-Tolyloxyacetic acid ethyl ester (3 a)**: To a solution of *o*-cresol (525 μL, 5.0 mmol, 1.0 equiv) in DMF (15 mL) under N_2_ was added NaH (300 mg, 7.5 mmol, 1.5 equiv). The reaction was allowed to stir for 60 min at RT under N_2_. To this was added ethyl bromoacetate (566 μL, 5.0 mmol, 1.0 equiv), and the reaction mixture was allowed to stir for 30 min. The reaction mixture was poured into cold H_2_O (25 mL) and allowed to stand for 60 min. No precipitate was observed. The aqueous phase was extracted with EtOAc, and the EtOAc layer was dried (Na_2_SO_4_) and concentrated under reduced pressure to give product **3 a** as a pale-yellow oil (740 mg, 76 % yield): *R*_f_=0.44 (EtOAc/petroleum ether (PE), 1:6); ^1^H NMR (400 MHz, [D_6_]DMSO): *δ*=7.20–7.08 (2 H, m, Ar-H), 6.86 (1 H, td, *J*=0.8, 7.4 Hz, Ar-H), 6.82 (1 H, d, *J*=8.2 Hz, Ar-H), 4.78 (2 H, s, CH_2_), 4.16 (2 H, q, *J*=7.1 Hz, *CH*_2_-CH_3_), 2.20 (3 H, s, CH_3_), 1.21 ppm (3 H, t, *J*=7.1 Hz, CH_2_-*CH*_3_); ^13^C NMR (100.6 MHz, [D_6_]DMSO): Cq: *δ*=168.8, 155.8, 126; CH: 130.6, 126.8, 120.9, 111.4; CH_2_: 64.9, 60.5; CH_3_: 16.0, 14.0 ppm; HRMS (ES): *m*/*z* [*M*+H]^+^ calcd for C_11_H_15_O_3_: 195.0943, found: 195.0962.

***p*****-Tolylaminoacetic acid ethyl ester (3 b)**: To a solution of *p*-toluidine (8.573 g, 80.0 mmol, 1.0 equiv) in EtOH (100 mL) was added acetic acid sodium salt (9.844 g, 120.0 mmol, 1.5 equiv) and heated at 90 °C for 5 min. To this was added ethyl bromoacetate (9.052 mL, 80.0 mmol, 1.0 equiv) and the reaction mixture was heated at reflux for 30 min. The reaction was cooled and concentrated under reduced pressure. The resulting residue was diluted with H_2_O and extracted with EtOAc. The EtOAc layer was then dried (Na_2_SO_4_) and concentrated under reduced pressure to result in a reddish-yellow oil. This was slowly turned to a pale-yellow solid under vacuum followed by recrystallisation from EtOH to furnish product **3 b** as a pale-yellow crystalline solid (2.360 g, 16 % yield): *R*_f_=0.25 (EtOAc/PE, 1:6); mp: 49–51 °C; ^1^H NMR (400 MHz, [D_6_]DMSO): *δ*=6.88 (2 H, d, *J*=8.1 Hz, Ar-H), 6.45 (2 H, d, *J*=8.4 Hz, Ar-H), 5.73 (1 H, t, *J*=6.4 Hz, NH), 4.10 (2 H, q, *J*=7.1 Hz, *CH*_2_CH_3_), 3.83 (2 H, d, *J*=6.5 Hz, CH_2_), 2.12 (3 H, s, CH_3_), 1.19 ppm (3 H, t, *J*=7.1 Hz, CH_2_*CH*_3_); ^13^C NMR (100.6 MHz, [D_6_]DMSO): Cq: *δ*=171.4, 145.8, 124.7; CH: 129.2, 112.2; CH_2_: 60.1, 45.0; CH_3_: 20.0, 14.1 ppm; HRMS (ES): *m*/*z* [*M*+H]^+^ calcd for C_11_H_16_NO_2_: 194.1103, found: 194.1119.

**(4-Methoxyphenylamino)acetic acid ethyl ester (3 c)**: To a solution of *p*-anisidine (9.951 g, 80.0 mmol, 1.0 equiv) in EtOH (150 mL) was added Et_3_N (13.380 mL, 96.0 mmol, 1.2 equiv), and the reaction mixture was stirred for 10 min. To this was added ethyl bromoacetate (9.052 mL, 80.0 mmol, 1.0 equiv), and the reaction mixture was heated at reflux for 75 min. The reaction was cooled and concentrated under reduced pressure. The resulting residue was diluted with H_2_O and extracted with EtOAc. Then EtOAc layer was dried (Na_2_SO_4_) and concentrated under reduced pressure to give a reddish powder, which was further washed with Et_2_O/PE (2:1) to afford product **3 c** as a pale-white powder (4.565 g, 27 % yield): *R*_f_=0.28 (EtOAc/PE, 1:3); mp: 58–60 °C; ^1^H NMR (400 MHz, [D_6_]DMSO): *δ*=6.71 (2 H, d, *J*=8.9 Hz, Ar-H), 6.50 (2 H, d, *J*=8.9 Hz, Ar-H), 5.53 (1 H, t, *J*=6.5 Hz, NH), 4.10 (2 H, q, *J*=7.1 Hz, *CH*_2_CH_3_), 3.81 (2 H, d, *J*=6.5 Hz, CH_2_), 3.63 (3 H, s, O*CH*_3_), 1.19 ppm (3 H, t, *J*=7.1 Hz, CH_2_*CH*_3_); ^13^C NMR (100.6 MHz, [D_6_]DMSO): Cq: *δ*=171.5, 151.1, 142.2; CH: 114.5, 113.2; CH_2_: 60.2, 45.5; CH_3_: 55.3, 14.1 ppm; HRMS (ES): *m*/*z* [*M*+H]^+^ calcd for C_11_H_16_NO_3_: 210.1052, found: 210.1320.

***o*****-Tolyloxyacetic acid hydrazide (4 a)**: To a solution of *o*-tolyloxyacetic acid ethyl ester (**3 a**, 657 mg, 3.38 mmol, 1.0 equiv) in EtOH (20 mL), a large excess of hydrazine (939 μL, 20.3 mmol, 6.0 equiv) was added, and the reaction mixture was heated at reflux overnight. The reaction mixture was cooled and concentrated under reduced pressure. The white precipitate obtained was washed with EtOH/PE (1:3) to afford product **4 a** as a white crystalline solid (367 mg, 60 % yield): *R*_f_=0.10 (EtOAc); mp: 114–116 °C; ^1^H NMR (400 MHz, [D_6_]DMSO): *δ*=9.20 (1 H, s, NH), 7.18–7.06 (2 H, m, Ar-H), 6.89–6.79 (2 H, m, Ar-H), 4.49 (2 H, s, CH_2_), 4.33 (2 H, bs, NH_2_), 2.21 ppm (3 H, s, CH_3_); ^13^C NMR (100.6 MHz, [D_6_]DMSO): Cq: *δ*=166.8, 156.0, 126.2; CH: 130.5, 126.8, 120.8, 111.3; CH_2_: 66.5; CH_3_: 16.1 ppm; HRMS (ES): *m*/*z* [*M*+H]^+^ calcd for C_9_H_13_N_2_O_2_: 181.0899, found: 181.1532.

***p*****-Tolylaminoacetic acid hydrazide (4 b)**: The procedure was similar to the procedure for **4 a** except that *p*-tolylaminoacetic acid ethyl ester (**3 b**, 1.930 g, 10.0 mmol, 1.0 equiv) was used and the reaction mixture and was heated at reflux for only 5 h. Product **4 b** was isolated as a pale-white powder (1.34 g, 75 % yield): *R*_f_=0.23 (EtOAc/MeOH, 9:1); mp: 151–153 °C; ^1^H NMR (400 MHz, [D_6_]DMSO): *δ*=9.00 (1 H, bs, *NH*-NH_2_), 6.89 (2 H, d, *J*=8.1 Hz, Ar-H), 6.46 (2 H, d, *J*=8.5 Hz, Ar-H), 5.59 (1 H, t, *J*=6.1 Hz, NH), 4.21 (2 H, bs, NH_2_), 3.58 (2 H, d, *J*=6.2 Hz, CH_2_), 2.14 ppm (3 H, s, CH_3_); ^13^C NMR (100.6 MHz, [D_6_]DMSO): Cq: *δ*=169.5, 146.0, 124.7; CH: 129.2, 112.4; CH_2_: 45.7; CH_3_: 20.0 ppm; HRMS (ES): *m*/*z* [*M*+H]^+^ calcd for C_9_H_14_N_3_O: 180.1059, found: 180.1158.

**(4-Methoxyphenylamino)acetic acid hydrazide (4 c)**: The procedure was similar to that for **4 a** except that (4-methoxyphenylamino)acetic acid ethyl ester (**3 c**, 5.0 g, 23.9 mmol, 1.00 equiv) was used in the reaction mixture. Product **4 c** was isolated as a white crystalline solid (3.986 g, 86 % yield): *R*_f_=0.20 (EtOAc/MeOH, 9:1); mp: 115–117 °C; ^1^H NMR (400 MHz, [D_6_]DMSO): *δ*=9.0 (1 H, bs, *NH*-NH_2_), 6.71 (2 H, d, *J*=8.9 Hz, Ar-H), 6.51 (2 H, d, *J*=8.9 Hz, Ar-H), 5.41 (1 H, t, *J*=6.2 Hz, NH), 4.21 (2 H, d, *J*=3.3 Hz, NH_2_), 3.63 (3 H, s, O*CH*_3_), 3.56 ppm (2 H, d, *J*=6.2 Hz, CH_2_); ^13^C NMR (100.6 MHz, [D_6_]DMSO): Cq: *δ*=170.1, 151.6, 142.9; CH: 115.0, 113.8; CH_2_: 46.7; CH_3_: 55.8 ppm; HRMS (ES): *m*/*z* [*M*+H]^+^ calcd for C_9_H_14_N_3_O_2_: 196.1008, found: 196.1473.

**4-(2-Ethylphenyl)-5-[(4-methoxyphenylamino)methyl]-4*H*-[1,2,4]triazole-3-thiol (6 a)**: To a solution of (4-methoxyphenylamino)acetic acid hydrazide (**4 c**) (4.0 g, 20.489 mmol, 1.0 equiv) in EtOH (150 mL) was added 2-ethylphenyl isothiocyanate (3.203 mL, 20.489 mmol, 1.0 equiv), and the reaction mixture was heated under reflux overnight. The reaction was cooled and concentrated under reduced pressure to result in a greasy yellow substance. To this was added aqueous 1.5 m KOH (50 mL) and heated at reflux for 30 min, then cooled. The reaction was neutralised with 5 n HCl and extracted with EtOAc. The organic layer was dried (Na_2_SO_4_) and concentrated under reduced pressure. The resulting yellow solid was recrystallised from EtOH/MeOH (1:1) to afford the desired product **6 a** as a white powder (2.246 g, 32 % yield): *R*_f_=0.65 (EtOAc); mp: 162–164 °C; ^1^H NMR (400 MHz, [D_6_]DMSO): *δ*=13.82 (1 H, s, SH or NH), 7.53–7.41 (2 H, m, Ar-H), 7.36 (1 H, td, *J*=1.6/1.7, 7.2/7.7 Hz, Ar-H), 7.27 (1 H, dd, *J*=1.2, 7.8 Hz, Ar-H), 6.67 (2 H, d, *J*=8.9 Hz, Ar-H), 6.45 (2 H, d, *J*=8.9 Hz, Ar-H), 5.40 (1 H, t, *J*=5.9/6.0 Hz, NH), 4.1–3.8 (2 H, m, CH_2_), 3.61 (3 H, s, OCH_3_), 2.4–2.2 (2 H, m, *CH*_2_-CH_3_), 1.07 ppm (3 H, t, *J*=7.6 Hz, CH_2_-*CH*_3_); ^13^C NMR (100.6 MHz, [D_6_]DMSO): Cq: *δ*=168.1, 151.3, 150.7, 141.8, 141.6, 131.9; CH: 130.1, 128.9, 128.6, 126.9, 114.4, 113.4; CH_2_: 39.3, 23.1; CH_3_: 55.2, 13.3 ppm; IR (KBr): 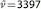 (NH), 3104, 3058 (CH of Ar), 2933, 2832 (CH from CH_2_), 1514 (C–N), 1308 cm^−1^ (C=S); HRMS (ES): *m*/*z* [*M*+H]^+^ calcd for C_18_H_21_N_4_OS: 341.1358, found: 340.9735.

**5-[(4-Methoxyphenylamino)methyl]-4-*p*-tolyl-4*H*-[1,2,4]triazole-3-thiol (6 b)**: To a solution of (4-methoxyphenylamino)acetic acid hydrazide (**4 c**) (2.0 g, 10.244 mmol, 1.0 equiv) in EtOH (100 mL) was added 4-methylphenyl isothiocyanate (1.575 g, 10.244 mmol, 1.0 equiv), and the reaction mixture was heated under reflux for 90 min, and the formation of precipitate was observed. The reaction was cooled, and the white precipitate formed was filtered and washed with EtOH. To the white precipitate was added aqueous 1.5 m KOH (20 mL) and heated at reflux for 30 min, then cooled. The reaction was neutralised with 5 n HCl and diluted with H_2_O followed by extraction with EtOAc. The organic layer was dried (Na_2_SO_4_) and concentrated under reduced pressure. The resulting pale-yellow solid was washed with EtOH/MeOH (1:1) to afford the desired product **6 b** as a pale-white powder (1.444 g, 43 % yield): *R*_f_=0.5 (EtOAc/PE, 3:1); mp: 158–160 °C; ^1^H NMR (400 MHz, [D_6_]DMSO): *δ*=13.76 (1 H, s, SH or NH), 7.34 (2 H, d, *J*=8.3 Hz, Ar-H), 7.30 (2 H, d, *J*=8.3 Hz, Ar-H), 6.67 (2 H, d, *J*=8.9 Hz, Ar-H), 6.45 (2 H, d, *J*=8.9 Hz, Ar-H), 5.42 (1 H, t, *J*=5.9/6.0 Hz, NH), 4.02 (2 H, d, *J*=6.0 Hz, CH_2_), 3.61 (3 H, s, OCH_3_), 2.38 ppm (3 H, s, CH_3_); ^13^C NMR (100.6 MHz, [D_6_]DMSO): Cq: *δ*=168.1, 151.2, 150.6, 141.7, 139.0, 130.9; CH: 129.8, 127.8, 114.4, 113.5; CH_2_: 39.4, CH_3_: 55.2, 20.8 ppm; HRMS (ES): *m*/*z* [*M*+H]^+^ calcd for C_17_H_19_N_4_OS: 327.1201, found: 326.9906.

**4-(4-Chlorophenyl)-5-(*p*-tolylaminomethyl)-4*H*-[1,2,4]triazole-3-thiol (6 c)**: *p*-Tolylaminoacetic acid hydrazide (**4 b**) (1.0 g, 5.581 mmol, 1.0 equiv) in EtOH (50 mL) was heated at 70 °C, resulting in a clear solution. To this was added 4-chlorophenyl isothiocyanate (947 mg, 5.58 mmol, 1.0 equiv), and the formation of a white precipitate was observed within 2 min. The reaction was allowed to stir for a further 10 min, and the reaction was cooled. The white precipitate formed was filtered and washed with EtOH. To the white precipitate was added aqueous 1.0 m NaOH (10 mL) and heated at reflux for 2 h, then cooled. Formation of a pale-white precipitate was observed. To this was added 25 mL cold H_2_O and neutralised with 5 n HCl. The resulting white solid was recrystallised with MeOH to afford the desired product **6 c** as a white crystalline solid (209 mg, 22 % yield): *R*_f_=0.68 (EtOAc); ^1^H NMR (400 MHz, [D_6_]DMSO): *δ*=7.54 (2 H, d, *J*=8.7 Hz, Ar-H), 7.39 (2 H, d, *J*=8.7 Hz, Ar-H), 6.83 (2 H, d, *J*=8.1 Hz, Ar-H), 6.41 (2 H, d, *J*=8.4 Hz, Ar-H), 5.53 (1 H, t, *J*=5.6 Hz, NH), 4.04 (2 H, d, *J*=5.6 Hz, CH_2_), 2.12 ppm (3 H, s, CH_3_); ^13^C NMR (100.6 MHz, [D_6_]DMSO): Cq: *δ*=167.8, 149.7, 145.5, 133.7, 133.2, 124.8; CH: 129.9, 129.1, 129.0, 112.4; CH_2_: 39.1; CH_3_: 20.0 ppm; HRMS (ES): *m*/*z* [*M*+H]^+^ calcd for C_16_H_16_ClN_4_S: 331.0706, found: 331.0877.

**4-Phenyl-5-*o*-tolyloxymethyl-4*H*-[1,2,4]triazole-3-thiol (6 d)**: To a solution of (**4 a**) *o*-tolyloxyacetic acid hydrazide (2.0 g, 11.095 mmol, 1.0 equiv) in EtOH (100 mL) was added phenyl isothiocyanate (1.356 mL, 11.095 mmol, 1.0 equiv), and the reaction mixture was heated under reflux for overnight, and the formation of precipitate was observed. The reaction was cooled, and the white precipitate formed was filtered and washed with EtOH. To the white precipitate was added aqueous 1.5 m KOH (20 mL) and heated at reflux for 30 min, then cooled. The reaction was neutralised with 5 n HCl. The white precipitate obtained was separated by filtration and washed further with H_2_O and freeze dried (2.093 g, 64 % yield): *R*_f_=0.30 (EtOAc/PE, 3:1) mp: 217–219 °C; ^1^H NMR (400 MHz, [D_6_]DMSO): *δ*=14.07 (1 H, s, SH or NH), 7.60–7.40 (5 H, m, Ar-H), 7.12–7.00 (2 H, m, Ar-H), 6.88 (1 H, d, *J*=8.0 Hz, Ar-H), 6.82 (1 H, td, *J*=0.4/0.5, 7.3/7.4 Hz, Ar-H), 5.01 (2 H, s, CH_2_), 1.85 ppm (3 H, s, CH_3_); ^13^C NMR (100.6 MHz, [D_6_]DMSO): Cq: *δ*=168.7, 155.1, 148.1, 133.5, 126.0; CH: 130.6, 129.5, 129.3, 127.9, 126.8, 121.2, 111.5; CH_2_: 59.9; CH_3_: 15.6 ppm; HRMS (ES): *m*/*z* [*M*+H]^+^ calcd for C_16_H_16_N_3_OS: 298.0936, found: 298.1035.

**4-(2-Ethylphenyl)-5-*o*-tolyloxymethyl-4*H*-[1,2,4]triazole-3-thiol (6 e)**: To a solution of (**4 a**) *o*-Tolyloxyacetic acid hydrazide (4.0 g, 22.19 mmol, 1.0 equiv) in EtOH (100 mL) was added 2-ethylphenyl isothiocyanate (3.470 mL, 22.19 mmol, 1.0 equiv), and the reaction mixture was heated under reflux overnight. The reaction was cooled and concentrated under reduced pressure, resulting in a white powder, which was further washed with EtOH/PE (1:3). To the white powder was added aqueous 1.5 m KOH (20 mL) and heated at reflux for 30 min, then cooled. The reaction was neutralised with 5 n HCl. The white precipitate obtained was separated by filtration and washed further with H_2_O and freeze dried (6.151 g, 85 % yield): *R*_f_=0.25 (EtOAc/PE, 1:3); mp: 212–214 °C; ^1^H NMR (400 MHz, [D_6_]DMSO): *δ*=14.12 (1 H, s, SH or NH), 7.53–7.40 (2 H, m, Ar-H), 7.39–7.27 (2 H, m, Ar-H), 7.12–7.03 (2 H, m, Ar-H), 6.90–6.78 (2 H, m, Ar-H), 4.89 (2 H, dd, *J*=13.0, 31 Hz, CH_2_), 2.48–2.23 (2 H, 2 hex, H_A_ & H_B_ of *CH*_2_CH_3_), 1.90 (3 H, s, CH_3_), 1.09 ppm (3 H, t, *J*=7.6, CH_2_-*CH*_3_); ^13^C NMR (100.6 MHz, [D_6_]DMSO): Cq: *δ*=168.6, 155.2, 148.2, 141.5, 131.8, 125.9; CH: 130.6, 130.2, 128.6, 128.6, 126.9, 121.2, 111.5; CH_2_: 59.8, 23.0; CH_3_: 15.6, 12.9 ppm; HRMS (ES): *m*/*z* [*M*+H]^+^ calcd for C_18_H_20_N_3_OS: 326.1249, found: 326.1287.

**4-(2-Chlorophenyl)-5-*o*-tolyloxymethyl-4*H*-[1,2,4]triazole-3-thiol (6 f)**: To a solution of (**4 a**) *o*-tolyloxyacetic acid hydrazide (2.0 g, 11.09 mmol, 1.0 equiv) in EtOH (100 mL) was added 2-chlorophenyl isothiocyanate (1.477 mL, 11.09 mmol, 1.0 equiv), and the reaction mixture was heated under reflux for 10 min; the formation of precipitate was observed. The reaction was cooled, and the white precipitate formed was filtered and washed with EtOH. To the white precipitate was added aqueous 1.5 m KOH (30 mL) and heated at reflux for 30 min, then cooled. The reaction was neutralised with 5 n HCl. The white precipitate obtained was separated by filtration and washed further with H_2_O and freeze dried (3.076 g, 84 % yield): *R*_f_=0.57 (EtOAc/PE, 1:1); mp: 156–158 °C; ^1^H NMR (400 MHz, [D_6_]DMSO): *δ*=14.16 (1 H, s, SH or NH), 7.73–7.66 (1 H, m, Ar-H), 7.62–7.47 (3 H, m, Ar-H), 7.12–7.02 (2 H, m, Ar-H), 6.89 (1 H, d, *J*=7.9 Hz, Ar-H), 6.82 (1 H, td, *J*=0.7, 7.4 Hz, Ar-H), 4.96 (2 H, dd, *J*=13.1, 29.7 Hz, CH_2_), 1.90 ppm (3 H, s, CH_3_); ^13^C NMR (100.6 MHz, [D_6_]DMSO): Cq: *δ*=168.8, 155.1, 147.9, 132.2, 140.0, 125.8; CH: 131.8, 130.7, 130.6, 130.2, 128.4, 126.8, 121.2, 111.3; CH_2_: 59.8; CH_3_: 15.6 ppm; HRMS (ES): *m*/*z* [*M*+H]^+^ calcd for C_16_H_15_ClN_3_OS: 332.0546, found: 332.0588.

**4-(2-Methoxyethyl)-5-*o*-tolyloxymethyl-4*H*-[1,2,4]triazole-3-thiol (6 g)**: To a solution of (**4 a**) *o*-tolyloxyacetic acid hydrazide (2.0 g, 11.09 mmol, 1.0 equiv) in EtOH (100 mL) was added 2-methoxyethyl isothiocyanate (1.203 mL, 11.09 mmol, 1.0 equiv), and the reaction mixture was heated under reflux overnight. The reaction was cooled, and the white precipitate formed was filtered and washed with EtOH. To the white precipitate was added aqueous 1.5 m KOH (30 mL) and heated at reflux for 30 min, then cooled. The reaction was neutralised with 5 n HCl. The white precipitate obtained was separated by filtration and washed further with H_2_O and freeze dried (2.787 g, 90 % yield): *R*_f_=0.13 (EtOAc/PE, 1:3); mp: 162–164 °C; ^1^H NMR (400 MHz, [D_6_]DMSO): *δ*=13.91 (1 H, s, SH or NH), 7.22–7.14 (2 H, m, Ar-H), 7.07 (1 H, d, *J*=8.0 Hz, Ar-H), 6.90 (1 H, td, *J*=0.8, 7.3 Hz, Ar-H), 5.21 (2 H, s, CH_2_), 4.21 (2 H, t, *J*=5.5 Hz, CH_2_), 3.62 (2 H, t, *J*=5.5 Hz, CH_2_), 3.22 (3 H, s, OCH_3_), 2.15 ppm (3 H, s, CH_3_); ^13^C NMR (100.6 MHz, [D_6_]DMSO): Cq: *δ*=167.4, 155.4, 148.7, 126.0; CH: 130.7, 127.0, 121.3, 111.8; CH_2_: 68.6, 60.1, 43.4; CH_3_: 58.4, 15.9 ppm; HRMS (ES): *m*/*z* [*M*+H]^+^ calcd for C_13_H_18_N_3_O_2_S: 280.1041, found: 280.1116.

**4-(4-Chlorophenyl)-5-*o*-tolyloxymethyl-4*H*-[1,2,4]triazole-3-thiol (6 h)**: To a solution of (**4 a**) *o*-tolyloxyacetic acid hydrazide (2.5 g, 13.872 mmol, 1.0 equiv) in EtOH (100 mL) at 80 °C was added 4-chlorophenyl isothiocyanate (2.376 g, 13.872 mmol, 1.0 equiv), and almost immediately formation of the precipitate was observed. The reaction mixture was heated under reflux for 10 min, and the reaction was cooled. The white precipitate formed was filtered and washed with EtOH. To the white precipitate was added aqueous 1.5 m KOH (47 mL) and heated at reflux for 30 min, then cooled. The reaction was neutralised with 5 n HCl. The white precipitate obtained was separated by filtration and washed further with distilled H_2_O and freeze dried (4.095 g, 89 % yield): *R*_f_=0.20 (EtOAc/PE, 1:3); mp: 152–154 °C; ^1^H NMR (400 MHz, [D_6_]DMSO): *δ*=14.11 (1 H, s, SH or NH), 7.60 (2 H, d, *J*=8.8 Hz, Ar-H), 7.51 (2 H, d, *J*=8.8 Hz, Ar-H), 7.14–7.03 (2 H, m, Ar-H), 6.90 (1 H, d, *J*=7.9 Hz, Ar-H), 6.83 (1 H, td, *J*=0.7, 7.4 Hz, Ar-H), 5.04 (2 H, s, CH_2_), 1.85 ppm (3 H, s, CH_3_); ^13^C NMR (100.6 MHz, [D_6_]DMSO): Cq: *δ*=168.7, 155.0, 148.1, 134.2, 132.5, 125.9; CH: 130.6, 129.9, 129.3, 126.8, 121.2, 111.6; CH_2_: 59.9; CH_3_: 15.5 ppm; HRMS (ES): *m*/*z* [*M*+H]^+^ calcd for C_16_H_15_ClN_3_OS: 332.0546, found: 332.0685.

**4-*p*-Tolyl-5-*o*-tolyloxymethyl-4*H*-[1,2,4]triazole-3-thiol (6 i)**: To a solution of (**4 a**) *o*-tolyloxyacetic acid hydrazide (2.5 g, 13.872 mmol, 1.0 equiv) in EtOH (100 mL) was added 4-methylphenyl isothiocyanate (2.134 g, 13.872 mmol, 1.0 equiv), and the reaction mixture was heated under reflux for 1 h; the formation of precipitate was observed. The reaction was cooled, and the white precipitate formed was filtered, washed with EtOH. To the white precipitate was added aqueous 1.5 m KOH (30 mL) and heated at reflux for 30 min, then cooled. The reaction was neutralised with 5 n HCl. The white precipitate obtained was separated by filtration and washed further with H_2_O and freeze dried (3.631 g, 84 %): *R*_f_=0.17 (EtOAc/PE, 1:3) mp: 177–179 °C; ^1^H NMR (400 MHz, [D_6_]DMSO): *δ*=14.04 (1 H, s, SH or NH), 7.35–7.26 (4 H, m, Ar-H), 7.04–7.12 (2 H, m, Ar-H), 6.88 (1 H, d, *J*=7.9 Hz, Ar-H), 6.83 (1 H, td, *J*=0.6, 7.4 Hz, Ar-H), 4.97 (2 H, s, CH_2_), 2.35 (3 H, s, CH_3_), 1.90 ppm (3 H, s, CH_3_); ^13^C NMR (100.6 MHz, [D_6_]DMSO): Cq: *δ*=168.7, 155.1, 148.2, 139.2, 130.9, 126.0; CH: 130.6, 129.7, 127.7, 126.9, 121.2, 111.6; CH_2_: 59.9; CH_3_: 20.7, 15.7 ppm; HRMS (ES): *m*/*z* [*M*+H]^+^ calcd for C_17_H_18_N_3_OS: 312.1092, found: 312.1252.

**4-(4-Methoxyphenyl)-5-*o*-tolyloxymethyl-4*H*-[1,2,4]triazole-3-thiol (6 j)**: To a solution of (**4 a**) *o*-tolyloxyacetic acid hydrazide (2.138 g, 11.86 mmol, 1.0 equiv) in EtOH (100 mL) was added 4-methoxyphenyl isothiocyanate (1.690 mL, 11.86 mmol, 1.0 equiv), and the reaction mixture was heated under reflux for 5 h. The reaction was cooled, and the white precipitate formed was filtered, washed with EtOH. To the white precipitate was added aqueous 1.5 m KOH (30 mL) and heated at reflux for 30 min, then cooled. The reaction was neutralised with 5 n HCl. The white precipitate obtained was separated by filtration and washed further with H_2_O and freeze dried (1.238 g, 32 % yield): *R*_f_=0.18 (EtOAc/PE, 1:1); mp: 155–157 °C; ^1^H NMR (400 MHz, [D_6_]DMSO): *δ*=14.02 (1 H, s, SH or NH), 7.36 (2 H, d, *J*=9.0 Hz, Ar-H), 7.12–7.02 (4 H, m, Ar-H), 6.90 (1 H, d, *J*=7.9 Hz, Ar-H), 6.87–6.80 (1 H, m, Ar-H), 4.97 (2 H, s, CH_2_), 3.79 (3 H, s, OCH_3_), 1.92 ppm (3 H, s, CH_3_); ^13^C NMR (100.6 MHz, [D_6_]DMSO): Cq: *δ*=168.9, 159.7, 155.2, 148.4, 126.0; CH: 130.6, 129.2, 126.9, 121.2, 114.4, 111.6; CH_2_: 59.9; CH_3_: 55.5, 15.7 ppm; HRMS (ES): *m*/*z* [*M*+H]^+^ calcd for C_17_H_18_N_3_O_2_S: 328.1041, found: 328.1217.

**4-(4-Isopropylphenyl)-5-*o*-tolyloxymethyl-4*H*-[1,2,4]triazole-3-thiol (6 k)**: The procedure was similar to that for **6 j**, except that 4-isopropylphenyl isothiocyanate (2.00 mL, 11.095 mmol, 1.0 equiv) was used. Product **6 k** was isolated as a white powder (1.914 g, 51 % yield): *R*_f_=0.61 (EtOAc/PE, 1:1); mp: 168–170 °C; ^1^H NMR (400 MHz, [D_6_]DMSO): *δ*=14.04 (1 H, s, SH or NH), 7.44–7.32 (4 H, m, Ar-H), 7.11–7.00 (2 H, m, Ar-H), 6.86 (1 H, d, *J*=7.9 Hz, Ar-H), 6.82 (1 H, td, *J*=0.7, 7.3 Hz, Ar-H), 5.03 (2 H, s, CH_2_), 3.04–2.84 (1 H, hept, CH of isopropyl), 1.81 (3 H, s, CH_3_), 1.21 ppm (6 H, d, *J*=6.9 Hz, (CH_3_)_2_ of isopropyl); ^13^C NMR (100.6 MHz, [D_6_]DMSO): Cq: *δ*=168.6, 155.1, 149.6, 148.3, 131.2, 126.1; CH: 130.5, 127.5, 127.1, 126.8, 121.2, 111.7, 33.2; CH_2_: 60.0; CH_3_: 23.7, 15.5 ppm; HRMS (ES): *m*/*z* [*M*+H]^+^ calcd for C_19_H_22_N_3_OS: 340.1405, found: 340.1575.

**Library synthesis**: The analogues of 1,2,4-triazole target compounds (**1 b**–**c**, **7 a**–**i** except **7 h**) were synthesised using a 12-well Radley’s parallel synthesiser.

**2-(4-Phenyl-5-*o*-tolyloxymethyl-4*H*-[1,2,4]triazol-3-ylsulfanyl)acetamide (7 a)**: A solution of product **6 d** (446 mg, 1.5 mmol, 1.0 equiv) in DMF (4 mL), and a solution of 2-bromoacetamide (211 mg, 1.5 mmol, 1.0 equiv) in DMF (2 mL) were both added separately to a dry Radley’s reaction tube containing K_2_CO_3_ (249 mg, 1.8 mmol, 1.2 equiv). The reaction mixture was allowed to stir at 45 °C overnight in a Radley’s parallel synthesiser. The reaction mixture was poured onto crushed ice (50 mL) and allowed to stand for ∼3 h. The precipitate formed was collected by filtration, washed thoroughly with H_2_O to remove DMF then freeze dried, followed by recrystallisation from EtOH/Et_2_O. Product **7 a** was obtained as a pale-yellow crystalline solid (232 mg, 44 % yield): *R*_f_=0.28 (EtOAc/MeOH, 9:1); mp: 123–125 °C; ^1^H NMR (400 MHz, [D_6_]DMSO): *δ*=7.68 (1 H, bs OH), 7.60–7.46 (5 H, m, Ar-H), 7.25 (1 H, bs, NH), 7.12–7.02 (2 H, m, Ar-H), 6.96 (1 H, d, *J*=7.8 Hz, Ar-H), 6.81 (1 H, td, *J*=0.8, 7.3/7.4 Hz, Ar-H), 5.13 (2 H, s, CH_2_), 3.95 (2 H, s CH_2_), 1.82 ppm (3 H, s, CH_3_); ^13^C NMR (100.6 MHz, [D_6_]DMSO): Cq: *δ*=168.5, 155.3, 151.8, 151.7, 132.8, 125.9; CH: 130.5, 130.0, 129.8, 126.8, 126.8, 121.0, 111.6; CH_2_: 59.7, 35.9; CH_3_: 15.6 ppm; HRMS (ES): *m*/*z* [*M*+H]^+^ calcd for C_18_H_19_N_4_O_2_S: 355.1150, found: 355.1118.

**2-[4-(2-Ethylphenyl)-5-*o*-tolyloxymethyl-4*H*-[1,2,4]triazol-3-ylsulfanyl]acetamide (1 b)**: The procedure was similar to that for **7 a** except that product **6 e** (325.5 mg, 1.0 mmol, 1.0 equiv) was used and the recrystallisation was carried out from EtOH. Product **1 b** was isolated as a white powder (147 mg, 39 % yield): *R*_f_=0.34 (EtOAc/MeOH, 9:1); mp: 134–136 °C; ^1^H NMR (400 MHz, [D_6_]DMSO): *δ*=7.68 (1 H, bs, OH), 7.58–7.46 (3 H, m, Ar-H), 7.43–7.37 (1 H, m, Ar-H), 7.34 (1 H, dd, *J*=1.4, 7.7 Hz; Ar-H), 7.24 (1 H, bs,=NH), 7.12–7.04 (2 H, m, Ar-H), 6.95 (1 H, d, *J*=7.9 Hz, Ar-H), 6.81 (1 H, td, *J*=0.8, 7.3/7.4 Hz, Ar-H), 5.00 (2 H, dd, *J*=12.7, 41.9 Hz, CH_2_), 3.97 (2H d, *J*=2.0 Hz, CH_2_), 2.33–2.18 (2 H, m, *CH*_2_CH_3_), 1.89 (3 H, s, CH_3_), 1.06 ppm (3 H, t, *J*=7.5 Hz, CH_2_*CH*_3_); ^13^C NMR (100.6 MHz, [D_6_]DMSO): Cq: *δ*=168.4, 155.5, 152.3, 151.7, 140.9, 125.8; CH: 130.9, 130.6, 129.1, 128.2, 127.3, 126.9, 121.1, 111.5; CH_2_: 59.6, 35.7, 22.7; CH_3_: 15.7, 13.3 ppm; HRMS (ES): *m*/*z* [*M*+H]^+^ calcd for C_20_H_23_N_4_O_2_S: 383.1463, found: 383.1430.

**2-[4-(2-Chlorophenyl)-5-*o*-tolyloxymethyl-4*H*-[1,2,4]triazol-3-ylsulfanyl]acetamide (7 b)**: The procedure was similar to that for **7 a** except that product **6 f** (332 mg, 1.0 mmol, 1.0 equiv) was used, and the precipitate obtained was washed with EtOH. Product **7 b** was isolated as a white crystalline solid (105 mg, 26 % yield): *R*_f_=0.30 (EtOAc/MeOH, 9:1); mp: 139–141 °C; ^1^H NMR (400 MHz, [D_6_]DMSO): *δ*=7.75 (1 H, dd, *J*=1.4, 8.0 Hz, Ar-H), 7.70–7.65 (2 H, m, OH & Ar-H), 7.61 (1 H, td, *J*=1.8/1.9, 7.6/7.8 Hz, Ar-H), 7.55 (1 H, td, *J*=1.5, 7.6 Hz, Ar-H), 7.26 (1 H, bs,=NH), 7.12–7.02 (2 H, m, Ar-H), 6.96 (1 H, d, *J*=7.7 Hz, Ar-H), 6.80 (1 H, td, *J*=0.8, 7.3 Hz, Ar-H), 5.10 (2 H, dd, *J*=12.9, 36.8 Hz, CH_2_), 3.95 (2 H, dd, *J*=14.9, 22.0 Hz, CH_2_), 1.85 ppm (3 H, s, CH_3_); ^13^C NMR (100.6 MHz, [D_6_]DMSO): Cq: *δ*=168.3, 155.3, 152.1, 151.6, 131.4, 130.2, 125.8; CH: 132.3, 130.5, 130.5, 130.0, 128.7, 126.8, 121.0, 111.3; CH_2_: 59.7, 36.3; CH_3_: 15.6 ppm; HRMS (ES): *m*/*z* [*M*+H]^+^ calcd for C_18_H_18_ClN_4_O_2_S: 389.0761, found: 389.0720.

**2-[4-(2-Methoxyethyl)-5-*o*-tolyloxymethyl-4*H*-[1,2,4]triazol-3-ylsulfanyl]acetamide (7 c)**: The procedure was similar to the procedure for **7 a** except that product **6 g** (419 mg, 1.5 mmol, 1.0 equiv) was used and the precipitate obtained was washed with EtOH/MeOH (5:1). Product **7 c** was isolated as a white powder (325 mg, 64 % yield): *R*_f_=0.22 (EtOAc/MeOH, 9:1); mp: 170–172 °C; ^1^H NMR (400 MHz, [D_6_]DMSO): *δ*=7.66 (1 H, bs, OH), 7.22 (1 H, bs,=NH), 7.21–7.10 (3 H, m, Ar-H), 6.92–6.86 (1 H, m, Ar-H), 5.27 (2 H, s, CH_2_), 4.24 (2 H, t, *J*=5.3 Hz, CH_2_), 3.91 (2 H, s, CH_2_), 3.60 (2 H, t, *J*=5.3 Hz, CH_2_), 3.20 (3 H, s, O*CH*_3_), 2.14 ppm (3 H, s, CH_3_); ^13^C NMR (100.6 MHz, [D_6_]DMSO): Cq: *δ*=168.7, 155.6, 151.9, 151.3, 125.9; CH: 130.7, 127.0, 121.1, 111.9; CH_2_: 70.1, 60.1, 43.8, 36.9; CH_3_: 58.4, 16.0 ppm; HRMS (ES): *m*/*z* [*M*+H]^+^ calcd for C_15_H_21_N_4_O_3_S: 337.1256, found: 337.1235.

**2-(4-*p*-Tolyl-5-*o*-tolyloxymethyl-4*H*-[1,2,4]triazol-3-ylsulfanyl)acetamide (7 d)**: The procedure was similar to that for **7 a** except that product **6 i** (467 mg, 1.5 mmol, 1.0 equiv) was used and the recrystallisation was carried out from EtOH. Product **7 d** was isolated as a white needle-like crystalline solid (224 mg, 41 % yield): *R*_f_=0.28 (EtOAc/MeOH, 9:1); mp: 136–138 °C; ^1^H NMR (400 MHz, [D_6_]DMSO): *δ*=7.68 (1 H, bs, OH), 7.41–7.33 (4 H, m, Ar-H), 7.24 (1 H, bs,=NH), 7.14–7.02 (2 H, m, Ar-H), 6.96 (1 H, d, *J*=7.9 Hz, Ar-H), 6.82 (1 H, td, *J*=0.8, 7.3 Hz, Ar-H), 5.09 (2 H, s, CH_2_), 3.94 (2 H, s, CH_2_), 2.36 (3 H, s, *CH*_3_), 1.88 ppm (3 H, s, CH_3_); ^13^C NMR (100.6 MHz, [D_6_]DMSO): Cq: *δ*=168.5, 155.4, 152.0, 151.7, 139.9, 130.1, 125.9; CH: 130.5, 130.2, 126.9, 126.6, 121.0, 111.6; CH_2_: 59.6, 35.9; CH_3_: 20.7, 15.7 ppm; HRMS (ES): *m*/*z* [*M*+H]^+^ calcd for C_19_H_21_N_4_O_2_S: 369.1307, found: 369.1411.

**2-[4-(4-Chlorophenyl)-5-*o*-tolyloxymethyl-4*H*-[1,2,4]triazol-3-ylsulfanyl]acetamide (7 e)**: The procedure was similar to that for **7 a** except that product **6 h** (497.7 mg, 1.5 mmol, 1.0 equiv) was used and the precipitate obtained was washed with EtOH/MeOH (3:1). Product **7 e** was isolated as a white crystalline solid (481 mg, 83 % yield): *R*_f_=0.26 (EtOAc/MeOH, 9:1); mp: 191–193 °C; ^1^H NMR (400 MHz, [D_6_]DMSO): *δ*=7.67 (1 H, bs, OH), 7.65 (2 H, d, *J*=8.8 Hz, Ar-H), 7.57 (2 H, d, *J*=8.8 Hz, Ar-H), 7.25 (1 H, bs,=NH), 7.13–7.03 (2 H, m, Ar-H), 6.97 (1 H, d, *J*=7.9 Hz, Ar-H), 6.82 (1 H, td, *J*=0.6, 7.3/7.4 Hz, Ar-H), 5.15 (2 H, s, CH_2_), 3.94 (2 H, s, CH_2_), 1.84 ppm (3 H, s, CH_3_); ^13^C NMR (100.6 MHz, [D_6_]DMSO): Cq: *δ*=168.4, 155.3, 151.8, 151.7, 134.7, 131.7, 125.8; CH: 130.5, 129.8, 128.8, 126.9, 121.1, 111.6; CH_2_: 59.7, 36.1; CH_3_: 15.6 ppm; HRMS (ES): *m*/*z* [*M*+H]^+^ calcd for C_18_H_18_ClN_4_O_2_S: 389.0761, found: 389.0978.

**2-[4-(4-Methoxyphenyl)-5-*o*-tolyloxymethyl-4*H*-[1,2,4]triazol-3-ylsulfanyl]acetamide (7 f)**: The procedure was similar to that for **7 a** except that product **6 j** (491 mg, 1.5 mmol, 1.0 equiv) was used and the recrystallisation was carried out from EtOH. Product **7 f** was isolated as a white powder (135 mg, 24 % yield): *R*_f_=0.24 (EtOAc/MeOH, 9:1); mp: 135–137 °C; ^1^H NMR (400 MHz, [D_6_]DMSO): *δ*=7.68 (1 H, bs, OH), 7.41 (2 H, d, *J*=9.0 Hz, Ar-H), 7.24 (1 H, bs,=NH), 7.13–7.04 (4 H, m, Ar-H), 6.97 (1 H, d, *J*=7.8 Hz, Ar-H), 6.82 (1 H, td, *J*=0.7, 7.3 Hz, Ar-H), 5.08 (2 H, s, CH_2_), 3.93 (2 H, s, CH_2_), 3.80 (3 H, s, O*CH*_3_), 1.90 ppm (3 H, s, CH_3_); ^13^C NMR (100.6 MHz, [D_6_]DMSO): Cq: *δ*=168.5, 160.1, 155.4, 152.3, 151.9, 125.9, 125.1; CH: 130.5, 128.3, 126.9, 121.0, 114.9, 111.6; CH_2_: 59.7, 35.8; CH_3_: 55.6, 15.8 ppm; HRMS (ES): *m*/*z* [*M*+H]^+^ calcd for C_19_H_21_N_4_O_3_S: 385.1256, found: 385.1466.

**2-[4-(4-Isopropylphenyl)-5-*o*-tolyloxymethyl-4*H*-[1,2,4]triazol-3-ylsulfanyl]acetamide (7 g)**: The procedure was similar to that for **7 a** except that product **6 k** (339.5 mg, 1.0 mmol, 1.0 equiv) was used and the recrystallisation was carried out from EtOH/Et_2_O. Product **7 g** was isolated as a white crystalline solid (300 mg, 76 % yield): *R*_f_=0.30 (EtOAc/MeOH, 9:1); mp: 135–137 °C; ^1^H NMR (400 MHz, [D_6_]DMSO): *δ*=7.69 (1 H, bs, OH), 7.47–7.36 (4 H, m, Ar-H), 7.25 (1 H, bs,=NH), 7.02–7.12 (2 H, m, Ar-H), 6.95 (1 H, d, *J*=7.8 Hz, Ar-H), 6.81 (1 H, td, *J*=0.8, 7.3/7.4 Hz, Ar-H), 5.14 (2 H, s, CH_2_), 3.95 (2 H, s, CH_2_), 2.96 (1 H, hep, CH of isopropyl), 1.80 (3 H, s, CH_3_), 1.21 ppm (6 H, d, *J*=6.9 Hz, (CH_3_)_2_ of isopropyl); ^13^C NMR (100.6 MHz, [D_6_]DMSO): Cq: *δ*=168.5, 155.3, 151.9, 151.8, 150.3, 126.0; CH: 130.5, 127.6, 126.8, 126.5, 121.0, 111.7, 33.2; CH_2_: 59.8, 35.9; CH_3_: 23.6, 15.6 ppm; HRMS (ES): *m*/*z* [*M*+H]^+^ calcd for C_21_H_25_N_4_O_2_S: 397.1620, found: 397.1510.

**2-[4-(4-Chlorophenyl)-5-(*p*-tolylaminomethyl)-4*H*-[1,2,4]triazol-3-ylsulfanyl]acetamide (7 h)**: To a solution of 4-(4-chlorophenyl)-5-(*p*-tolylaminomethyl)-4*H*-[1,2,4]triazole-3-thiol (**6 c**; 200 mg, 0.604 mmol, 1.0 equiv) in acetone (7 mL) was added added 2-bromoacetamide (83.0 mg, 0.604 mmol, 1.0 equiv) and K_2_CO_3_ (100 mg, 0.725 mmol, 1.2 equiv). The reaction mixture was allowed to stir at 45 °C for 4 h. The reaction mixture was evaporated under reduced pressure. The residue was diluted with H_2_O and extracted with EtOAc. Then EtOAc layer was dried (Na_2_SO_4_) and concentrated under reduced pressure. Purification of the residue by column chromatography [CH_2_Cl_2_/MeOH (5 %)] gave product **7 h** as a pale-yellow crystalline solid (41 mg, 17 % yield): *R*_f_=0.19 (EtOAc/MeOH, 9:1); mp: 170–172 °C; ^1^H NMR (400 MHz, [D_6_]DMSO): *δ*=7.66 (1 H, bs, OH), 7.63 (2 H, d, *J*=8.7 Hz, Ar-H), 7.48 (2 H, d, *J*=8.7 Hz, Ar-H), 7.23 (1 H, bs,=NH), 6.83 (2 H, d, *J*=8.2 Hz, Ar-H), 6.42 (2 H, d, *J*=8.4 Hz, Ar-H), 5.65 (1 H, t, *J*=5.6 Hz, NH), 4.19 (2 H, d, *J*=5.6 Hz, CH_2_), 3.88 (2 H, s, CH_2_), 2.12 ppm (3 H, s, CH_3_); ^13^C NMR (100.6 MHz, [D_6_]DMSO): Cq: *δ*=168.5, 154.0, 150.6, 145.6, 134.6, 131.8, 124.9; CH: 129.8, 129.2, 129.0, 112.4; CH_2_: 38.4, 36.1; CH_3_: 20.1 ppm; HRMS (ES): *m*/*z* [*M*+H]^+^ calcd for C_18_H_19_ClN_5_OS: 388.0921, found: 388.1184.

**2-[5-[(4-Methoxyphenylamino)methyl]-4-*p*-tolyl-4*H*-[1,2,4]triazol-3-ylsulfanyl]acetamide (1 c)**: The procedure was similar to that for **7 a** except that product **6 b** (200 mg, 0.612 mmol, 1.0 equiv) was used and the recrystallisation was carried out from EtOH. Product **1 c** was isolated as a white powder (147 mg, 63 % yield): *R*_f_=0.16 (EtOAc/MeOH, 9:1); mp: 157–159 °C; ^1^H NMR (400 MHz, [D_6_]DMSO): *δ*=7.65 (1 H, bs, OH), 7.37 (2 H, d, *J*=8.2 Hz, Ar-H), 7.33 (2 H, d, *J*=8.5 Hz, Ar-H), 7.21 (1 H, bs,=NH), 6.66 (2 H, d, *J*=8.9 Hz, Ar-H), 6.49 (2 H, d, *J*=9.0 Hz, Ar-H), 5.44 (1 H, t, *J*=5.7 Hz, NH), 4.13 (2 H, d, *J*=5.7 Hz, CH_2_), 3.88 (2 H, s, CH_2_), 3.61 (3 H, s, O*CH*_3_), 2.39 ppm (3 H, s, CH_3_); ^13^C NMR (100.6 MHz, [D_6_]DMSO): Cq: *δ*=168.6, 154.0, 151.2, 150.7, 142.1, 139.7, 130.3; CH: 130.2, 126.8, 114.4, 113.5; CH_2_: 38.9, 35.9; CH_3_: 55.3, 20.8 ppm; HRMS (ES): *m*/*z* [*M*+H]^+^ calcd for C_19_H_22_N_5_O_2_S: 384.1416, found: 384.1509.

**2-[4-(2-Ethylphenyl)-5-[(4-methoxyphenylamino)methyl]-4*H*-[1,2,4]triazol-3-ylsulfanyl]acetamide (7 i)**: The procedure was similar to that for **7 a** except that product **6 a** (340.5 mg, 1.0 mmol, 1.0 equiv) was used and the recrystallisation was carried out from EtOH. Product **7 i** was isolated as a white crystalline solid (222 mg, 56 % yield): *R*_f_=0.3 (EtOAc/MeOH, 9:1); mp: 134–136 °C; ^1^H NMR (400 MHz, [D_6_]DMSO): *δ*=7.65 (1 H, bs, OH), 7.57–7.46 (4 H, m, Ar-H), 7.21 (1 H, bs,=NH), 6.65 (2 H, d, *J*=9.0 Hz, Ar-H), 6.48 (2 H, d, *J*=9.0 Hz, Ar-H), 5.40 (1 H, t, *J*=5.7/5.8 Hz, NH), 3.91 (2 H, d, *J*=1.9 Hz, CH_2_), 3.61 (3 H, s, O*CH*_3_), 2.21 (2 H, q, *J*=7.6 Hz, -*CH*_2_CH_3_), 1.03 ppm (3 H, t, *J*=7.5 Hz, -CH_2_*CH*_3_); ^13^C NMR (100.6 MHz, [D_6_]DMSO): Cq: *δ*=168.5, 154.1, 151.2, 151.0, 142.1, 141.1, 131.0; CH: 130.6, 129.3, 128.1, 127.2, 114.4, 113.4; CH_2_: 38.9, 35.7, 22.8; CH_3_: 55.2, 13.6 ppm; IR (KBr): 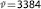 (NH), 3154 (CH of Ar), 2977, 2831 (CH from CH_2_), 1666 (C–O), 1517 cm^−1^(C–N); HRMS (ES): *m*/*z* [*M*+H]^+^ calcd for C_20_H_24_N_5_O_2_S: 398.1572, found: 398.1344.
